# Protective Role of *AMPK* against *PINK1*^*B9*^ Flies' Neurodegeneration with Improved Mitochondrial Function

**DOI:** 10.1155/2023/4422484

**Published:** 2023-10-12

**Authors:** Guoliang Xiang, Xueyi Wen, Wenjing Wang, Tianchan Peng, Jiazhen Wang, Qinghua Li, Junfang Teng, Ying Cui

**Affiliations:** ^1^Department of Neurology Intensive Care Unit, The First Affiliated Hospital of Zhengzhou University, Zhengzhou, Henan 450052, China; ^2^Department of Neurology, Affiliated Hospital of Guilin Medical University, Guilin, Guangxi 541004, China; ^3^Department of Neurology and Stroke Center, The First Affiliated Hospital of Jinan University, Guangzhou 510630, China; ^4^Department of Neurology, Xiangya Hospital of Central South University, Changsha, Hunan 410008, China; ^5^Department of Neurology, The First Affiliated Hospital of Zhengzhou University, Zhengzhou, Henan 450052, China

## Abstract

Adenosine 5′-monophosphate-activated protein kinase (*AMPK*)'s effect in *PTEN*-induced kinase 1 (*PINK1*) mutant Parkinson's disease (PD) transgenic flies and the related mechanism is seldom studied. The classic *MHC-Gal4/UAS* PD transgenic flies was utilized to generate the disease characteristics specifically expressed in flies' muscles, and Western blot (WB) was used to measure the expression of the activated form of *AMPK* to investigate whether activated *AMPK* alters in *PINK1*^*B9*^ PD flies. *MHC-Gal4* was used to drive *AMPK* overexpression in *PINK1*^*B9*^ flies to demonstrate the crucial role of *AMPK* in PD pathogenesis. The abnormal wing posture and climbing ability of *PINK1*^*B9*^ PD transgenic flies were recorded. Mitochondrial morphology via transmission electron microscopy (TEM) and ATP and NADH: ubiquinone oxidoreductase core subunit S3 (NDUFS3) protein levels were tested to evaluate the alteration of the mitochondrial function in *PINK1*^*B9*^ PD flies. Phosphorylated AMPK*α* dropped significantly in *PINK1*^*B9*^ flies compared to controls, and *AMPK* overexpression rescued *PINK*^*B9*^ flies' abnormal wing posture rate. The elevated dopaminergic neuron number in PPL1 via immunofluorescent staining was observed. Mitochondrial dysfunction in *PINK1*^*B9*^ flies has been ameliorated with increased ATP level, restored mitochondrial morphology in muscle, and increased NDUFS3 protein expression. Conclusively, *AMPK* overexpression could partially rescue the PD flies via improving *PINK1*^*B9*^ flies' mitochondrial function.

## 1. Introduction

PD symptoms are the result derived from dopamine, dopaminergic, DA selectivity in the two areas of the brain induced by dopamine neurons loss in the substantia nigra compacta (substantia nigra pars compacta, SNc) and striatum degeneration [1, 2]. The other feature of PD exists in surviving neurons with a synapse nucleoprotein (*α*-synuclein) as the main composition of the formation of Lewy bodies (LB) [3, 4]. Sporadic forms of PD are usually due to oxidative stress caused by environmental toxins, such as rotenone, N, N′-dimethyl-4,4′-bipyridinium dichloride (paraquat), and 1-methyl-4-phenyl-1, 2, 3, 6-tetrahydropyridine (MPTP), and these substances target mitochondrial electron transport complexes from which they impair mitochondrial function [5, 6]. Although the low incidence of sporadic PD is extremely low, recent studies found that many of the genes in familial PD plays an essential role in mitochondrial function, such as *α*-synuclein (*PARK1*), *UCHL1* (*PARK5*), *LRRK2* (*PARK8*) dominant mutation, *PINK1* (*PARK6*), *DJ-1* (*PARK7*) mutations, and *Parkin* (*PARK2*) [7]. PD can be classified into familial and sporadic types according to genetic pathogenesis, and gene mutations are more common in familial PD [8–10]. *PINK1* genes are considered one of the common pathogenic genes that lead to autosomal recessive PD [11–13]. *PINK1* gene encodes mitochondrial serine/threonine kinase associated with mitochondrial autophagy and quality control [14]. Therefore, *PINK1* mutations related to PD models that present with the obvious mitochondrial dysfunction-induced neurodegenerative disease have become a hot research issue. The vital role of mitochondria is to generate ATP as the energy core station as it is the most critical organelles in eukaryotes to meet the need for energy metabolism [15, 16]. The morphology of mitochondria is in a state of constant changes in response to the different metabolic situations [17, 18]. Under normal conditions, the fission and fusion of mitochondria are in a state of constant conversion, and *PINK1* gene mutation can disrupt the balance of such conversion [19, 20]. The disorder of mitochondrial quality control (MQC) is closely related to neurodegenerative diseases [21].

Numerous studies have indicated the flight muscle degeneration of *PINK1*^*B9*^ in the PD transgenic fruit fly model caused by the *PINK1* gene mutation resulting in flying capacity impairment, abnormal wing posture, significantly shortened life span, and significantly lowered dopamine neuron content than the normal control group [22, 23].

Mitochondrial dysfunction is the most obvious pathogenic characteristic in *PINK1*^*B9*^ flies, with hyperinflated and degenerated plaques of mitochondria in the flight muscle [24, 25]. Therefore, direct energy regulation of *PINK1*^*B9*^ flies might improve their mitochondrial function.

*AMPK* is a highly conserved energy sensor in eukaryotes that is capable of sensing metabolic stress [26, 27]. When energy balance is disturbed in cellular metabolism, *AMPK* activation induces alterations in metabolism of cells and regulates gene expression to decrease anabolic metabolism, promote catabolism, and recover ATP level [27]. *AMPK* is rapidly activated after nearly all mitochondrial stresses, even those that do not disrupt the mitochondrial membrane potential. *AMPK* regulates autophagy and mitophagy through the activation of the kinase *ULK1*, the mammalian homologue of *ATG1* [28]. *AMPK* phosphorylates mitochondrial fission factor and promotes mitochondrial fission upon energetic stress. By simultaneously regulating mitochondrial fission, mitophagy, and transcriptional control of mitochondrial biogenesis, *AMPK* acts as a signal integration platform to maintain mitochondrial health [29].

It has been found that deactivated *AMPK* exacerbates loss of neurons and associated phenotypes in *LRRK* and parkin mutant flies, while activated *AMPK* may lead a potential therapeutic clue for familial PD [30]. However, its role in *PINK1*-mutated PD models has not been studied.

Neurons mainly use glucose as the main energy source. While the brain is one of the most crucial organs in the body to absorb glucose, more than 50% of the total glucose is used; as a result, the neurons have extremely high energy demand, and however, they cannot store too much energy [31, 32]. Hence, the energy storage capacity and demand are crucial since neurons are sensitive to cellular energy fluctuations [33]. Therefore, *AMPK* keeps an essential function in the central nervous system (CNS) in maintaining the neurons' function and in maintaining homeostasis of cellular energy [34]. *AMPK* is the crucial kinase in regulating energy metabolism, which might be an important clue in *PINK1*-mutated PD pathogenesis. However, the function or regulation of *AMPK* in the *PINK1*-mutated PD model and its mechanism have not been studied.

This study is to investigate *AMPK*'s function in *PINK1*^*B9*^ PD transgenic flies, interfering *AMPK* expression for PD transgenic flies. Intervention was conducted to observe whether *AMPK* overexpression (*AMPK* OE) or RNA interference with *AMPK* (*AMPK* RNAi) had protective effects on PD transgenic flies and further explore the mechanism of such protective effects.

## 2. Materials and Methods

### 2.1. Drosophila Lines and Husbandry

All flies were treated in a 12-hour light-dark cycle with the standard cornmeal, agar medium, yeast at 50% relative humidity, 25°C. *MHC-Gal4* (muscle) and *TH-Gal4* (tyrosine hydroxylase) were used to construct the corresponding systems in this study for the tissue-specific genes' knockdowns and upregulation. The isogenic *W*^*1118*^ and *UAS*-*AMPK* OE (32108) were obtained from Bloomington Drosophila Stock Center. *PINK1*^*B9*^ fly line was a gift given by Dr. J Chung from KAIST, Korea. Separately, we used *MHC-Gal4* and *TH-Gal4* driver to cross with *W*^*1118*^ to generate the control flies. Only male flies were chosen from the day they hatched and were transferred to the new tubes within ten days.

### 2.2. Abnormal Wing Postures Quantification

One hundred male flies were chosen randomly from each experimental group and were put in the transparent tubes (25 cm in length and 1.6 cm in diameter) with putting 5 flies in each after anesthesia. Then, it was incubated at room temperature for 20 min to acclimate to the environment. Data obtained from more than three independent experiments are presented as mean ± S.D. of scores. At room temperature and under standard light conditions, all behavioral tests were performed.

### 2.3. Climbing Assay

Climbing assay was applied to evaluate locomotor function. One hundred flies from each indicated group were applied to in this assay for more than three independent experiments. Each fly was placed in a vertically placed tube (1.6 cm in diameter and 25 cm in length). The flies were tapped to the bottom of the tube. The total climbing time phase was collected by crossing over the 6 cm line from the tube bottom. Fly numbers were noted that could go over the 6 cm line within 10 seconds. The data were shown as the percentage of total flies in this experiment and were shown as average +SEM by more than three independent experiments. The statistical analysis was processed via two-way ANOVA (*p* < 0.05).

### 2.4. Transmission Electron Microscopy (TEM)

Ten-day-old fly thoraxes were prepared following fixing for 12 hours in 2.5% glutaraldehyde and 2% paraformaldehyde. A transmission electron microscope is used to examine the ultrathin sections. Ten thoraxes were selected from each experimental group. At Guangxi Medical University, Pathology Department, we collected the TEM images. At 10,000 x magnification, the slices were recorded by using the electron microscopy (H-7650, HITACHI, Japan). Mitochondrial perimeter was determined using ImageJ software.

### 2.5. Dopaminergic Neurons Immunostaining

PPL1 TH-positive neurons quantification was evaluated using whole-mounted brain samples immune-stained from each experimental group. Ten-day-old fly brain samples were dissected in cold phosphate-buffered saline (PBS). The brain samples were put in 0.3% Triton X-100 (Sigma) and 4% paraformaldehyde in PBS and set at 25°C for 1 hour. Then, the brains were kept incubated at 4°C overnight by antityrosine hydroxylase (TH) (1 : 500 diluted for use, Millipore, Boston, MA). The following day, the brains were washed in 0.3% Triton X-100 in PBS three times and each for 20 minutes at room temperature. Afterwards, brain samples were then incubated using fluorescent secondary antibody at 25°C for 1 hour. After mounting the samples, they were imaged via the confocal microscope (Wetzlar, Germany). PPL1 TH-positive neuron quantification was analyzed from the brain hemisphere.

### 2.6. ATP Content Measurement

Five thoraxes from each group were harvested and the tissue was washed in cold PBS. The tissue was homogenized in 100 *μ*L ice-cold 2N PCA with a homogenizer sitting on ice. Samples were centrifuged at 13,000 g for 2 minutes at 4°C in a cold centrifuge and the supernatant was transferred to a fresh tube. After homogenizing the tissue in 100 *μ*L of PCA, the volume was diluted to 500 *μ*L with the Assay Buffer XXIII/ATP Assay Buffer included in the ATP kit (ab83355). The ATP content of each group was extracted and was detected following the protocol of manufacturer and was detected by Ex/Em = 535/587 nm fluorometric to show mitochondrial functions for more than three independent experiments. Protein amount was standardized by protein assay (Bradford), while the abundance of ATP was analyzed according to the protein amount.

### 2.7. Reactive Oxygen Species (ROS) Level Detection

ROS was measured using the CellROX Orange reagent (cat. no. BB-470512; Shanghai Bio-Tech Co., Ltd.). The thoraxes of 30 Drosophila were obtained, homogenized, and centrifuged at 1,000 x g for 10 min at 4°C, and the supernatant was collected. The supernatant and 20 *μ*M CellROX Orange Reagent were mixed and incubated at 37°C in the dark for 30 min, and the fluorescence intensity at 510 and 610 nm (maximum excitation light and maximum emission wavelength) was measured using a multifunction microplate reader.

### 2.8. Respirometry Measurement

Using the high-resolution respirometer, Oxygraph-2K equipment, mitochondrial oxygen consumption was collected. Thorax tissues were isolated freshly, homogenized on ice, and placed in the reaction buffer (80 mmol/l KCl, 3 mmol/l magnesium chloride, 1 mmol/l EDTA, 10 mmol/l Tris/HCl, and 5 mmol/l potassium phosphate, pH 7.4). Then, inhibitors, substrates, and uncouplers were used as described below: substrates including 2M glutamate, 0.8M malate, and 2M pyruvate (P + M + G), 4 mM cytochrome C (Cyt-c), and 0.5M ADP^+^ Mg^2+^; uncoupler including rotenone and antimycin A (AMA); and 1.0 mM carbonyl cyanide m-chlorophenyl hydrazone (CCCP) for measuring mitochondrial respiratory chain complexes. Using malate, pyruvate, and glutamate as the substrates, complex I respiration was measured. Complex II was assessed via respiration buffer mixed with 10 mM succinate and 1 mM rotenone. Oxygen flux and oxygen concentration indicating CI and CII capacity were collected by Oroboros Instruments DatLab software for more than three independent experiments.

### 2.9. Western Blots (WB)

Ten-day-old fly thoraxes from ten flies each group were flash-frozen using liquid nitrogen followed by homogenizing using the pestle in 2x lysis buffer (2 mM EDTA; 300 mM NaCl; 2% Triton X-100; 1% SDS; 50 mM TRIS, pH 8.0) with Sigma protease inhibitor cocktail. The homogenates were centrifuged at the speed of 21,000 g for 10 minutes, and the supernatant was subjected to WB procedure. Anti-TH rabbit polyclonal (ab152) (Millipore, Billerica, MA, USA); anti-phospho-ULK1 (Ser467) (CST4634) (CST, Danvers, Massachusetts, USA); anti-p-AMPK-*α* (Thr172) (CST#2535); anti-AMPK-*α* (ab80039); anti-NDUFS3 mouse monoclonal (ab14711) (MitoSciences, Eugene, OR, USA); and anti-*α*-tubulin IgG1 clone B-5-1-2 (Sigma-Aldrich, St Louis, MO, USA) were used in this experiment. The secondary antibodies used were as follows: peroxidase conjugated anti-mouse (Abcam, Cambridge, MA) and peroxidase-conjugated anti-rabbit IgG (GE Healthcare, Piscataway, NJ or Jackson ImmunoResearch, West Grove, PA). Signals were tested by using SuperSignal West Pico Chemiluminescent Substrate (Thermo Scientific, Rockford, IL) or Pierce ECL Western Blotting Substrate. WB tests were conducted for more than three independent experiments.

### 2.10. RT-PCR

According to the instructions of manufacturer, total RNA was extracted by using TRI Reagent (Sigma-Aldrich) from ten flies' thorax. Dissolve the RNA pellet in 5 *μ*l RNAase-free water. RT-PCRs were processed more than three times by Life Technologies 7500 Real-Time PCR System with SYBR Green chemistry (Promega). The 2−ΔΔCt (RQ, relative quantification) quantification method was utilized. The following primers used in this study were as follows: 18s-forward: 5′-TCTAGCAATATGAGATTG AGCAATAAG-3′; 18s-reverse: 5′-AATACACGTTGATACTTTCATTGTAGC-3′; *AMPKα*-forward: 5′-TAAATAAGCAAAACGCGAAGAA-3′; *AMPKα*-reverse: 5′-CAAGGGCGGTTGAATTGATACT-3′.

Endogenous control used in this study was 18S.

### 2.11. Statistics

Values represented as means ± SEM. Data were calculated by GraphPad Prism 8 via either unpaired two-tailed Student's *t*-test or one-way ANOVA test to determine significance. ^*∗*^*p* < 0.05, ^*∗∗*^*p* < 0.01, ^*∗∗∗*^*p* < 0.005, and ^#^*p* < 0.001 were expressed in all figures to show the significant difference between other groups (NS, not significantly different).

## 3. Results

### 3.1. Pathogenic Features in *PINK1*^*B9*^ Flies

As previous studies presented, *PINK1*^*B9*^ flies presented the severely abnormal wing posture and climbing ability (Figures S1A–S1C) accompanied with the severely damaged mitochondrial morphology and the decreased mitochondrial number per observation field in flight muscle tested by TEM (Figures S1D–S1F). ATP level decreased dramatically in *PINK1*^*B9*^ compared to the W^1118^ flies (Figure S1G), and ROS (reactive oxygen species) increased significantly in *PINK1*^*B9*^ compared to the W^1118^ flies (Figure S1H) indicating the severely imbalanced energy metabolism. *MHC-Gal4* drove *AMPK OE* flies did not show significant difference compared to W^1118^ control flies (Figures S1A–S1H).

### 3.2. AMPK Alteration in *PINK1*^*B9*^ Flies

**Figure 1:**
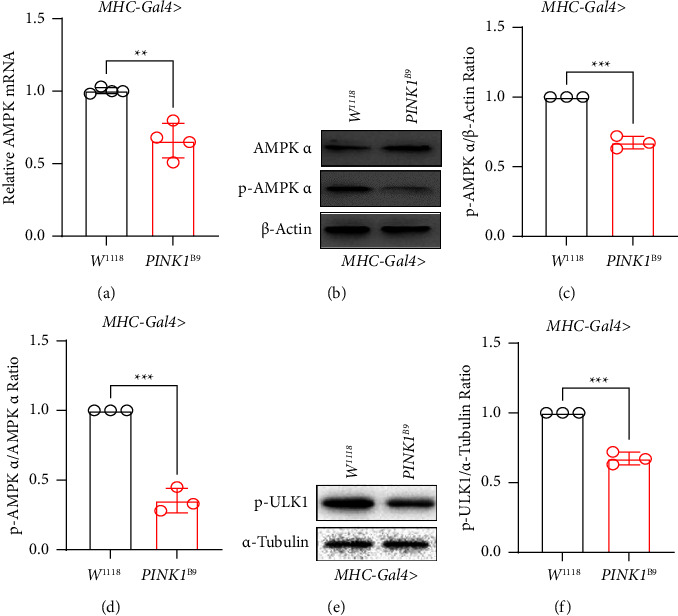
AMPK alteration in MHC-Gal4 system *PINK1*^*B9*^ flies. (a) Relative AMPK mRNA expression in each group (*n* ≥ 5/group) tested by RT-PCR. (b–d) p-AMPK-*α* and AMPK-*α* protein expression detected by WB in each group (*n* ≥ 10/group). *β*-Actin was used as loading control. (e, f) p-ULK1 protein expression detected by WB in each group (*n* ≥ 10/group). *α*-Tubulin was used as loading control. Values were expressed as mean ± S.D. (^*∗∗*^*P* < 0.01, ^*∗∗∗*^*P* < 0.001).

Here, we introduce *AMPK*, an enzyme essential for maintaining energy balance. Interestingly, the decreased *AMPK* mRNA expression was observed in *PINK1*^*B9*^ flies (Figure 1(a)) consistent with the decreased activation form of *AMPK*, phosphorylated *AMPK*-*α* (p-*AMPK*-*α*) tested by WB (Figures 1(b)–1(d)). To further confirm *AMPK* pathway alterations, p-ULK1 (phosphorylated unc-51-like autophagy-activating kinase 1) expression was detected which is regarded as the downstream of *AMPK*. With the same trend as p-AMPK-*α*, we observed the decreased p-ULK1 in *PINK1*^*B9*^ flies (Figures 1(e) and 1(f)) [35].

### 3.3. Construction of AMPK Interfered *PINK1*^*B9*^ Flies

**Figure 2:**
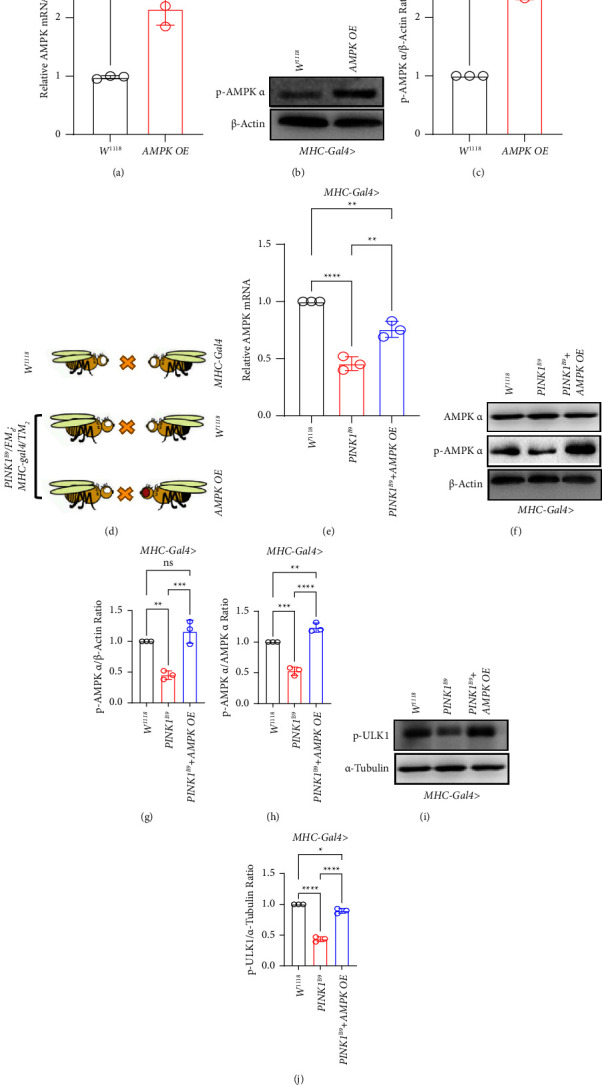
*AMPK* expression and target protein in AMPK pathway detected in *AMPK* overexpressed *PINK1*^*B9*^ flies. (a) *AMPK* mRNA expression confirmation in MHC-Gal4 drove *AMPK* interfered flies (*n* ≥ 3/group). (b, c) Immunoblot of the indicated genotypes of flies (*n* ≥ 10/group) with the antibody specific to the phosphorylated form of p-AMPK-*α*. *β*-Actin serves as a loading control. (d) Drosophila line generating strategy. (e–h) Immunoblot of the indicated genotypes of flies (*n* ≥ 10/group) with the antibody specific to the phosphorylated form of AMPK *α*, total-AMPK, and *β*-actin serves as a loading control. (i, j) Immunoblot of the indicated genotypes of flies (*n* ≥ 10/group) with the antibody specific to the phosphorylated form of ULK1 and *α*-tubulin serves as a loading control. Data are shown as the mean ± S.D. (^*∗*^*P* < 0.05, ^*∗∗*^*P* < 0.01, ^*∗∗∗*^*P* < 0.001, ^*∗∗∗∗*^*P* < 0.0001, ns = not significant).

*AMPK* OE and *AMPK* RNAi transgenic flies driven by the classical *MHC-Gal4* muscle promoter were verified by RT-PCR to confirm the correct *AMPK* expression alterations in the mRNA level compared with the normal control group (Figure S2A). The expression level of *AMPK* mRNA and p-AMPK-*α* was significantly higher in *AMPK* OE flies and lower in *AMPK* RNAi compared with the normal controls (Figures 2(a)–2(c) and S2A). The construction strategy of *AMPK* overexpressed *PINK1*^*B9*^ flies is shown in Figure 2(d). More importantly, as the increased expression of *AMPK* mRNA was examined in *AMPK OE PINK1*^*B9*^ flies, we confirmed the main activated form of *AMPK* protein expression, p-*AMPK*-*α* in *AMPK* overexpressed *PINK1*^*B9*^ flies from which we observed the highly induced p-AMPK-*α* protein expression in *AMPK* OE *PINK1*^*B9*^ flies compared with *PINK1*^*B9*^ flies (Figures 2(e)–2(h)). Accompanied by p-AMPK-*α* expression alteration in *AMPK* OE *PINK1*^*B9*^ flies, we observed the increased p-ULK1 expression in *AMPK* OE *PINK1*^*B9*^ flies (Figures 2(i) and 2(j)).

### 3.4. AMPK Overexpression Improves *PINK1*^*B9*^ Flies' Abnormal Wing Posture and Climbing Activity

**Figure 3:**
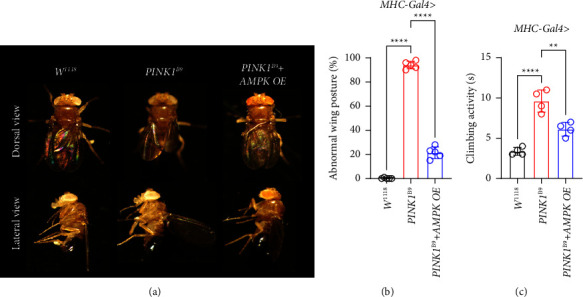
*AMPK* overexpression improves *PINK1*^*B9*^ flies' abnormal wing posture. (a) Wing posture phenotypes in the indicated genotypes of flies (*n* ≥ 5/group). (b) Abnormal wing posture tested in each group (*n* ≥ 100/group). (c) Climbing ability examined in each group (*n* ≥ 100/group). Values were expressed as mean ± S.D. (^*∗∗*^*P* < 0.01, ^*∗∗∗∗*^*P* < 0.0001).

Overexpression of *AMPK* expression has a protective effect on *PINK1*^*B9*^ flies since we observed the significant improvement in abnormal wing posture and climbing activity in *AMPK* OE *PINK1*^*B9*^ flies compared with *PINK1*^*B9*^ flies (Figures 3(a)–3(c)). However, we did not observe any improvement in *AMPK* RNAi interfered *PINK1*^*B9*^ flies (Fig. S2B). Hence, upregulated *AMPK* expression had a partial protective effect on *PINK1*^*B9*^ PD transgenic flies.

### 3.5. AMPK Overexpression Suppresses *PINK1*^*B9*^ Flies' Neurodegeneration

**Figure 4:**
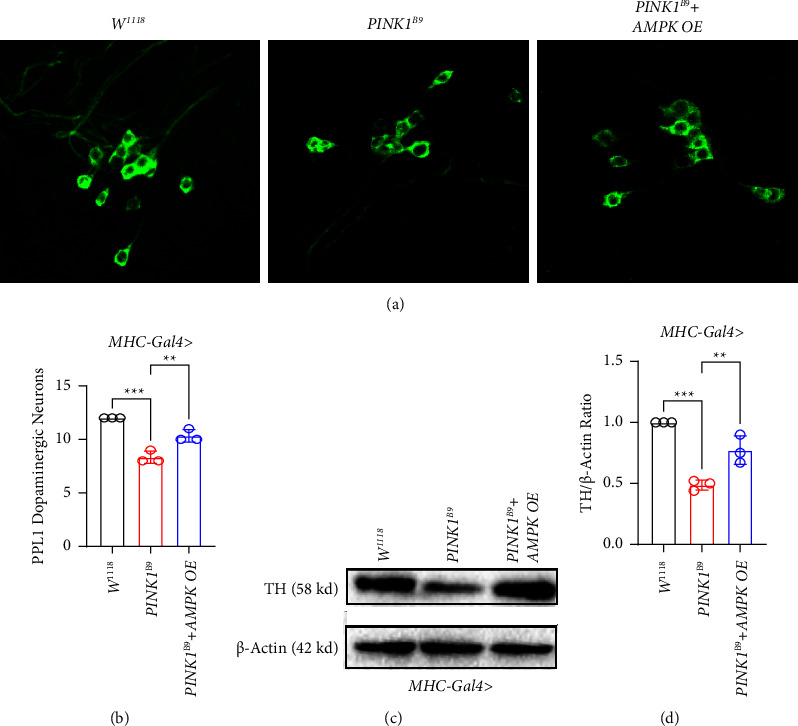
*AMPK* overexpression suppresses *PINK1*^*B9*^ flies' neurodegeneration. (a) Whole brains of 10-day-old male flies (*n* ≥ 5/group) driven by TH-gal4 of the indicated genotypes were stained with antityrosine hydroxylase (TH) antibody. Panel shows confocal images of DA neuron of the PPL1 cluster of the indicated genotypes. (b) Quantification of the PPL1 neurons in brains of the indicated genotypes (*n* ≥ 3/group) in each condition. Bar graph shows the number of PPL1 cluster dopaminergic neurons. (c) Whole-fly brain lysates (*n* ≥ 10/group) were analyzed by western blot analysis by using the tyrosine hydroxylase (TH) antibody. (d) Relative tyrosine hydroxylase (TH) protein expression in the brains (*n* ≥ 3/group). Values were expressed as mean ± S.D. (^*∗∗*^*P* < 0.01, ^*∗∗∗*^*P* < 0.001).

To further confirm whether *AMPK* overexpression directly affects *PINK1*^*B9*^ flies' pathogenesis, we detected PPL1 cluster degeneration with the dopaminergic neurons in *PINK1*^*B9*^ flies since it has been well demonstrated previously. We used the classic *TH-Gal4* transgene to modulate *AMPK* expression in *PINK1*^*B9*^ flies crossed with *AMPK* OE flies separately. Reorganization and characterization of midbrain DA neurons were conducted by the whole-mount immunostaining from which we observed a significantly increased cell body number in PPL1 of *AMPK* OE *PINK1*^*B9*^ flies compared to the *PINK1*^*B9*^ flies (Figures 4(a) and 4(b)). Moreover, TH protein expression levels were confirmed in *AMPK* overexpressed *PINK1*^*B9*^ via WB, indicating the increased TH protein expression in *AMPK* OE *PINK1*^*B9*^ flies compared with *PINK1*^*B9*^ flies (Figures 4(c) and 4(d)), which is consistent with the immunostaining results.

### 3.6. AMPK Overexpression Improves *PINK1*^*B9*^ Flies' Mitochondrial Function

**Figure 5:**
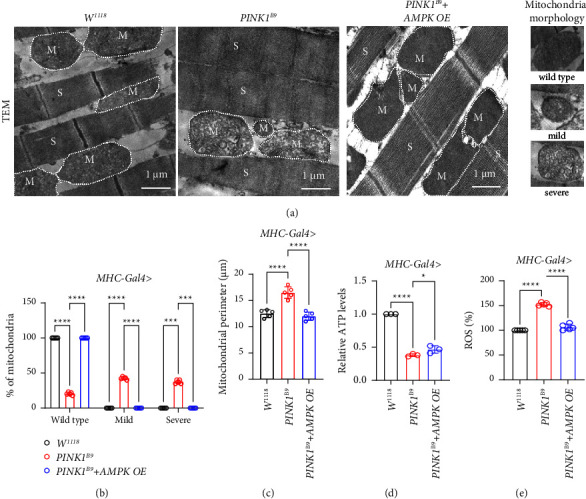
*AMPK* overexpression improves *PINK1*^*B9*^ flies' mitochondrial function. (a–c) Representative TEM images of adult flight muscles showing mitochondrial morphology (*n* ≥ 5/group). (d, e) Relative ATP and ROS levels tested in each group (*n* ≥ 5/group). Values were expressed as mean ± S.D. (^*∗*^*P* < 0.05, ^*∗∗∗*^*P* < 0.001, ^*∗∗∗∗*^*P* < 0.0001).

Since one of the pathogenic signatures of *PINK1*^*B9*^ flies is the defect of mitochondrial morphology accompanied with the impaired mitochondrial function in *MHC-Gal4* system, we further explored whether *AMPK* overexpression had any effect on *PINK1*^*B9*^ flies' flight muscle mitochondria. Surprisingly, the improved mitochondrial morphology was detected by thorax muscles' TEM sections in *AMPK* OE *PINK1*^*B9*^ flies with the more normally shaped mitochondrial morphology (Figures 5(a)–5(c)). In order to further confirm mitochondrial function in *AMPK* overexpressed *PINK1*^*B9*^ PD flies, we tested muscle tissue ATP and ROS levels separately in each group. Surprisingly, we found that ATP level increased significantly in *AMPK* OE *PINK1*^*B9*^ flies while ROS decreased significantly in *AMPK* OE *PINK1*^*B9*^ flies compared with the disease model (Figures 5(d) and 5(e)).

### 3.7. AMPK Overexpression Improves *PINK1*^*B9*^ Flies' CI Function of ETC

**Figure 6:**
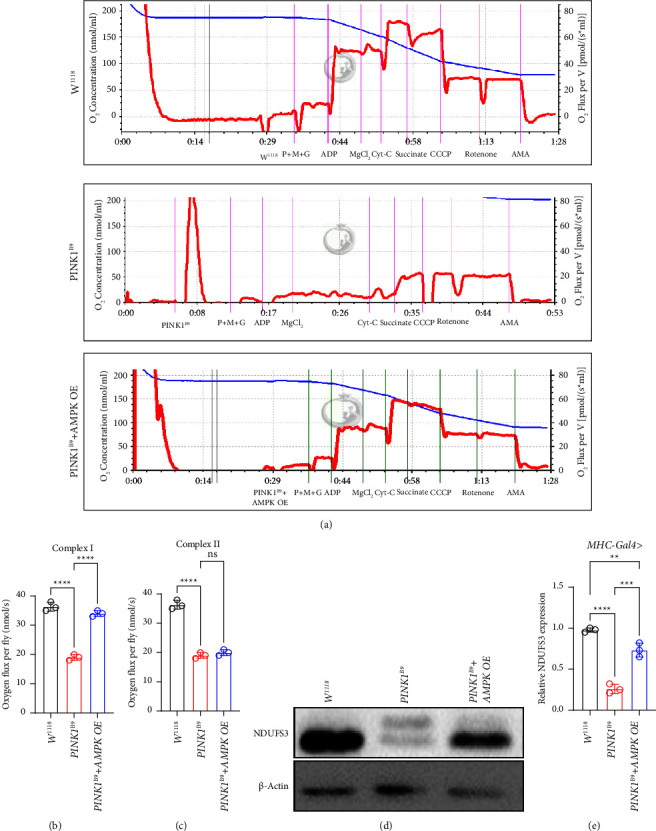
*AMPK* overexpression improves *PINK1*^*B9*^ flies' CI function of ETC. (a–c) Quantification of mitochondrial respiratory CI and CII function of ETC tested in thorax tissue (*n* ≥ 5 group). (d, e) Equal amounts of protein (80 *μ*g), isolated from fly thorax muscles of the indicated phenotype (*n* ≥ 10/group), were separated by SDS-PAGE and immunoblotted using the NDUFS3 antibody. Relative NDUFS3 protein expression in the thorax muscles of the indicated genotypes of flies (mean ± S.D.; ^*∗*^*P* < 0.05, ^#^*P* < 0.0001).

Since ETC complexes' enzyme activity and the different levels of ETC subunits expression are positively correlated with MQC. Moreover, among them, CI and CII assembly and activity are crucially important in maintaining ETC function in the mitochondrial matrix. To examine whether *AMPK* overexpression directly impacts mitochondrial ETC, we utilized the Oroboros O2k equipment to detect the mitochondrial respiratory chain's overall activity using the freshly isolated muscle from the indicated genotypes of flies homogenized on ice which is applied into the examination. Interestingly, we observed a significant increase in CI function in *MHC-Gal4*-driven *AMPK* OE interfered *PINK1*^*B9*^ flies compared with the *PINK1*^*B9*^ disease controls (Figures 6(a) and 6(b)). However, we did not see any improvement in CII function of ETC (Figures 6(a)–6(c)).

Here, we introduce nicotinamide adenine dinucleotide dehydrogenase (ubiquinone) Fe-S protein 3 (NDUFS3) which plays a crucial role in composing CI of mitochondria. To further test whether the content of NDUFS3 is consistent with CI function improvement in *AMPK* overexpressed *PINK1*^*B9*^ flies, we conducted WB using the muscle tissue via the same *MHC-Gal4*-driven system. As our expectation, NDUFS3 protein expression increased significantly in *AMPK* OE *PINK1*^*B9*^ flies (Figures 6(d) and 6(e)).

## 4. Discussion

PD is one of the most common seen neurodegenerative diseases with motor dysfunction. At present, PD affects about 5∼6 million people around the world. With the rapid growth of the world population, the number is expected to reach 10 million by 2030. The pathogenesis of PD is mainly due to aging and environmental factors, while the specific pathogenesis is still unclear. Dysfunction of mitochondria is increasingly regarded as a threatening factor in the susceptibility of PD dopaminergic neurons and has become the pathogenesis characteristic of familial PD. Recently, researchers have shown that *PINK1* gene mutation is one of the primary pathogenic genes causing familial PD, and *PINK1* protein has been proved to be a vital element for MQC [36].

Therefore, we concluded that in the mutant PD drosophila model, *PINK1*^*B9*^ flies, the indirect flight muscles were not orderly with the decreased dopaminergic neuron number in PPL1, and the number of mitochondrial number per observation field was reduced accompanied by the decreased ATP level, which directly reflecting mitochondrial function was significantly decreased consistently to previous studies [37].

*AMPK* is an energy sensor of cells, capable of sensing metabolic stress and integrating many physiological signaling pathways to restore energy balance [38]. Some studies have shown that abnormal energy metabolism is a risk factor associated with neurodegenerative diseases [39]. Furthermore, recent studies suggest that *AMPK* activation has a neuroprotective function [40]. *AMPK*'s activator, 5-amimidazole 4-formamide ribonucleotide (AICAR), protects hippocampal neurons by resisting glucose deficiency damage and glutamate toxicity and inhibits the apoptotic level of astrocytes induced by ceramide. At the same time, some studies have found that gallic acid can significantly inhibit dopamine and mitochondrial dysfunction with its effects depending on the activity of *AMPK*, as the protective effect of gallic acid is eliminated when *AMPK* activity is inhibited. Since numerous dietary supplements and pharmaceuticals (e.g., AICAR or metformin) that increase AMPK activity are available for use in humans which might slow down the disease progression of PD, the clinical studies of their effects in PD patients are limited. Recent studies indicated that when treated with AMPK activator, parkin and LRRK2 mutants significantly improve the DA neuronal and climbing phenotypes in Ddc-GAL4-LRRK2 G2019S flies [30].

So that in our study, regulate *AMPK* expression in PD transgenic flies, especially the overexpression of *AMPK* might rescue its neurodegeneration phenotypes. The experimental results showed that in the PD transgenic flies, the main *AMPK* activation form p-AMPK-*α* was significantly lower than normal controls. Thus, we speculated that the overexpression of *AMPK* in PD transgenic flies might rescue its phenotype and other disease features. As our expectation, *AMPK* OE can indeed improve PD transgenic flies' abnormal wing posture and the climbing ability. Consistently, dopaminergic neuron numbers increased significantly in *AMPK* OE interfered with *PINK1*^*B9*^ flies so that the upregulation of *AMPK* rescues PD transgenic flies' muscle mitochondrial morphology and improves the mitochondrial content per unit area. Also, *AMPK* overexpression improved *PINK1*^*B9*^ flies' muscle ATP content. Studies have shown that the substantia nigra striatum in PD disease models, mitochondrial respiratory chain CI, and the activity of the coenzyme Q10 declined [41]. Here, we found the increased CI subunit, NDUFS3 protein expression tested by WB, and the increased CI function in *AMPK* OE interfered PD.

This study used a classical model of PD flies verified increased activation of *AMPK* expression-protected *PINK1* mutant PD transgenic flies. The activity of *AMPK* enables the energy metabolism to reach a balanced state, thus finding a possible therapeutic target for neurodegenerative diseases treatment like PD.

Our study is the first to show that the expression level of p-*AMPK*-*α* protein, the main activated form of *AMPK*, was significantly decreased in *PINK1*^*B9*^ mutant PD Drosophila model compared with the normal control. After interfering by *AMPK* OE with the *PINK1*^*B9*^, the abnormal wing posture of the PD transgenic drosophila model could be restored significantly, while the expression level of the activated form of *AMPK* protein in *PINK1*^*B9*^ flies' muscle tissue increased dramatically.

When detecting *AMPK* OE interfered *PINK1*^*B9*^ phenotypes, we found that the abnormal wing posture rate decreased significantly and improved climbing ability with the increased dopaminergic neuron numbers in PPL1, indicating the protective role of *AMPK* on neurodegeneration of *PINK1*^*B9*^ flies. We also found the severely impaired mitochondrial function in the PD transgenic flies flight muscle was restored accompanied by the restored mitochondrial morphology, increased ATP levels, and the increased expression of the key marker of mitochondrial respiratory chain Complex I subunits and NDUFS3 protein. Furthermore, understanding overexpressed AMPK protects PINK1-mutated PD flies which might indicate a therapeutic target on PINK1 mutant form of PD.

## Data Availability

The data supporting the current study are available from the corresponding author upon request.

## Supplementary Materials

Supplementary Materials Supplementary File 1: pathogenic features in *PINK1*^*B9*^ flies. Supplementary File 2: *AMPK* alteration in *MHC-Gal4* system *PINK1*^*B9*^ flies. Fig. S1: pathogenic features in *PINK1*^*B9*^ flies. (A) Wing posture phenotypes in the indicated genotypes of flies (*n* ≥ 3/group). (B, C) Abnormal wing posture and flying ability tested in each group (*n* ≥ 100/group). (D–F) Representative TEM images of adult flight muscles showing mitochondrial morphology (*n* ≥ 4/group). (G, H) Relative ATP and ROS levels tested in each group (*n* ≥ 5/group). Values were expressed as mean ± S.D. (^*∗*^*P* < 0.05, ^*∗∗∗∗*^*P* < 0.0001, ns = not significant). Fig. S2: downregulation of AMPK did not rescue motor deficits of PINK1B9 flies. (A) Relative AMPK mRNA expression in each group (*n* ≥ 5/group). (B) Climbing ability examined in each group (*n* ≥ 100/group). Values were expressed as mean ± S.D. (^*∗∗*^*P* < 0.01, ^*∗∗∗∗*^*P* < 0.0001, ns = not significant).
