# Supplementary material for: Protective Role of AMPK against PINK1B9 Flies' Neurodegeneration with Improved Mitochondrial Function
Source: Parkinsons Dis. 2023 Oct 12;2023:4422484. doi: 10.1155/2023/4422484 (PMC10586901; doi:10.1155/2023/4422484)
Supplement: Supplementary Materials — Supplementary File 1: pathogenic features in PINK1B9 flies. Supplementary File 2: AMPK alteration in MHC-Gal4 system PINK1B9 flies. Fig. S1: pathogenic features in PINK1B9 flies. (A) Wing posture phenotypes in the indicated genotypes of flies (n ≥ 3/group). (B, C) Abnormal wing posture and flying ability tested in each group (n ≥ 100/group). (D–F) Representative TEM images of adult flight muscles showing mitochondrial morphology (n ≥ 4/group). (G, H) Relative ATP and ROS levels tested in each group (n ≥ 5/group). Values were expressed as mean ± S.D. (∗P < 0.05, ∗∗∗∗P < 0.0001, ns = not significant). Fig. S2: downregulation of AMPK did not rescue motor deficits of PINK1B9 flies. (A) Relative AMPK mRNA expression in each group (n ≥ 5/group). (B) Climbing ability examined in each group (n ≥ 100/group). Values were expressed as mean ± S.D. (∗∗P < 0.01, ∗∗∗∗P < 0.0001, ns = not significant). [file 4422484.f1.zip › Figures Guoliang Xiang et al V2 S1 (1).pdf]

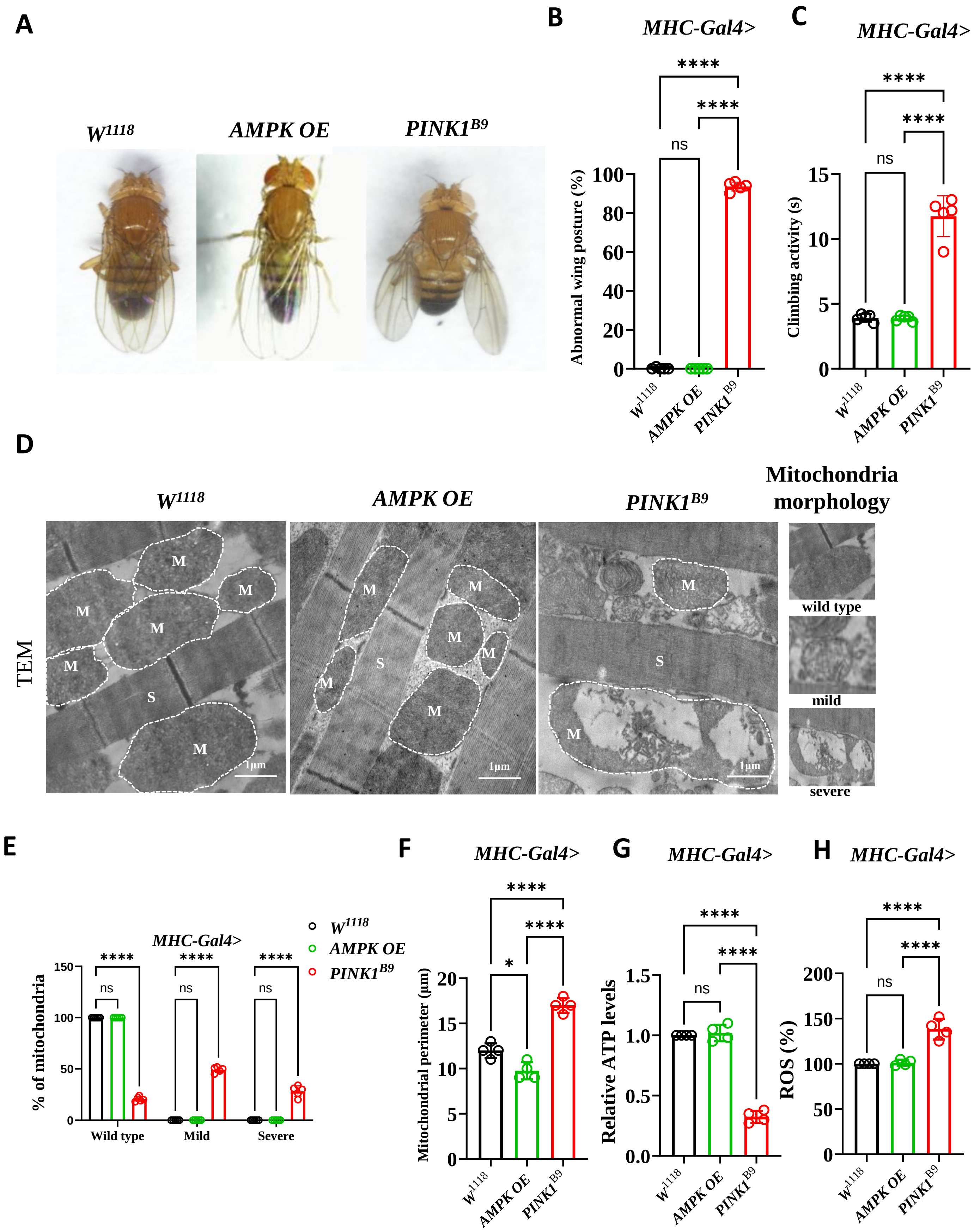

Fig. S1. Pathogenic features in *PINK1*^{B9} flies.
(A) Wing posture phenotypes in the indicated genotypes of flies. (n≥3/group) **(B)** and **(C)** Abnormal wing posture and flying ability tested in each group. (n≥100/group) **(D), (E) and (F)** Representative TEM images of adult flight muscles showing mitochondrial morphology. (n≥4/group) **(G) and (H)** Relative ATP and ROS levels tested in each group. (n≥5/group) Values were expressed as mean ± S.D. (*P<0.05, ****P<0.0001, ns=not significant).
